# The impact of Composite Dietary Antioxidant Index on the relationship between eczema and depression symptoms in US adults

**DOI:** 10.3389/fnut.2024.1470833

**Published:** 2024-11-22

**Authors:** Tian Zhang, Ruohua Zhang, Ting Song, Fang Chen, Yuegang Wei

**Affiliations:** Department of Dermatology, Affiliated Hospital of Nanjing University of Chinese Medicine, Jiangsu Province Hospital of Chinese Medicine, Nanjing, China

**Keywords:** Composite Dietary Antioxidant Index, eczema, depression, moderating, NHANES

## Abstract

**Aims:**

The study aims to explore the associations of eczema, Composite Dietary Antioxidant Index (CDAI), with depression symptoms in adults based on the National Health and Nutrition Examination Surveys (NHANES) database.

**Methods:**

In total, 3,402 participant data were extracted from the NHANES 2005–2006. The relationship between eczema, CDAI, and depression symptoms was explored by utilizing weighted univariate and multivariate logistic regression models, presenting as odds ratios (ORs) and 95% confidence intervals (CIs). The additive interaction between eczema, CDAI, and depression symptoms was measured by relative excess risk due to interaction (RERI) and the attributable proportion of interaction (AP). Subsequently, the associations of eczema, CDAI, with depression were also explored in different gender, body mass index (BMI), and smoking subgroups.

**Results:**

Of the 3,402 participants included, the mean age was 46.76 (0.83) years old, and 174 (5.11%) participants had depression symptoms. In the adjusted model, both eczema (OR = 3.60, 95%CI: 2.39–5.40) and CDAI (OR = 1.97, 95%CI: 1.19–3.27) were associated with a higher prevalence of depression symptoms. Compared to the participants with high CDAI and no eczema, those participants with low CDAI (eczema: OR = 7.30, 95%CI: 4.73–11.26; non-eczema: OR = 1.84, 95%CI: 1.06–3.19) have higher odds of depression symptoms, no matter have eczema or not. When under low CDAI levels, eczema was associated with increased odds of depression symptoms (OR = 3.76, 95%CI: 2.34–6.03). When under low CDAI level, eczema was also related to elevated odds of depression symptoms in those males, females, BMI <25, BMI ≥25, non-smoking, and smoking.

**Conclusion:**

CDAI could modulate the association of eczema with depression symptoms in adults.

## 1 Introduction

Eczema, or atopic dermatitis, is a chronic inflammatory skin condition characterized by pruritus, recurrent eczematous patches, and plaques (1). Typically manifesting in early childhood, it may persist into adulthood or even emerge later in life (1). In the United States (U.S), approximately 10–20% of adults experience eczema at some point (2, 3). Beyond the physical discomfort it causes, eczema profoundly impacts mental health (4). Adults with eczema are at an elevated risk of developing psychiatric disorders, including depression, anxiety, and sleep disturbances (5, 6). The burden of eczema transcends its dermatological symptoms, resulting a significant reduction in quality of life and increased healthcare utilization due to both physical discomfort and associated psychological distress (4).

Depression, a common comorbidity of eczema, further exacerbates the considerable burden of the condition (7). Individuals with eczema are at a heightened risk of experiencing depression compared to those without the disorder (8). The mechanisms underlying this association are complex and may involve chronic stress due to the physical symptoms of eczema, social stigma, sleep disturbances, and the psychological impact of living with a visible chronic condition (8–10). Understanding the relationship between eczema and depression is essential for developing effective interventions that address both the dermatological and psychological aspects of the disease.

Oxidative stress is a pivotal pathophysiological mechanism implicated in both eczema and depression (11, 12). The Composite Dietary Antioxidant Index (CDAI) is a comprehensive measure of dietary antioxidant intake, encompassing a broad spectrum of antioxidants, including vitamins C and E, carotenoids, and flavonoids (13). Research indicates that CDAI was inversely associated with depression in the general population (14). Additionally, dietary antioxidant intake has shown a negative correlation with childhood eczema (15). Given these associations, CDAI may serve as a potential moderator influencing the relationship between eczema and depression. Hence, this study aims to investigate the moderating effect of CDAI on the association between eczema and depression symptoms in adults.

## 2 Methods

### 2.1 Study design and participants

The National Health and Nutrition Examination Surveys (NHANES), a major program by the National Center for Health Statistics (NCHS), assesses the nutrition and health status of both adults and children in the U.S. The NHANES protocol has received approval from the NCHS Research Ethics Review Board, with all participants providing informed consent before the survey. The ethical approval was granted with an exemption from the Jiangsu Province Hospital of Chinese Medicine.

Data for participants were extracted from the NHANES 2005–2006 cycle, the only period with complete questionnaires on eczema. The participants included individuals aged ≥20 years old, who having an assessment for eczema and depression symptoms, as well as dietary intake data. Exclusion criteria comprised: (1) individuals taking antidepressants, (2) extreme caloric intake (defined as <600 kcal or > 4,200 kcal per day for men, and < 500 kcal or > 3,600 kcal per day for women) (16), and (3) missing data on important covariates.

### 2.2 Determination of eczema

Eczema diagnosis was based on self-reported data from the NHANES questionnaire, where individuals indicated a positive response to the query, “Has a doctor or other health professional ever told you that you have eczema” (17).

### 2.3 CDAI assessment

CDAI was calculated using six dietary antioxidant micronutrients: vitamins A, C, E, zinc, magnesium, and selenium, following Wright’s method (17). Each nutrient was standardized by subtracting the mean and dividing by the standard deviation to estimate CDAI. Standardized intakes were then summarized with equal weighting to derive the composite CDAI. In our study, the CDAI score was categorized into two levels based on the median.

### 2.4 Depression assessment

Depression symptoms were defined using the Patient Health Questionnaire (PHQ-9), a 9-item screening tool assessing the frequency of depressive symptoms over the past 2 weeks via face-to-face mobile examination center interview (18). Respondents rated symptoms such as anhedonia, depressed mood, sleep disturbance, fatigue, appetite changes, low self-esteem, concentration problems, psychomotor disturbances, and suicidal ideation on a 0–3 scale. The PHQ-9 total score ranges from 0 to 27 points, with a score ≥ 10 indicating clinically relevant depressive symptoms (19).

### 2.5 Covariates

The covariates in this study included race, poverty income ratio (PIR), marriage, education level, sleep duration, smoking, house smoker, hypertension, diabetes, cardiovascular disease (CVD), and serum vitamin D. Smoking status was determined according to the question “Have you smoked at least 100 cigarettes in your entire life.” Hypertension, as measured by NHANES, was self-reported, applying antihypertensive drugs and blood pressure measurements consistent with previous studies (20, 21). Self-reported hypertension was assessed by participants having a positive answer to the question “Have you ever been told by a doctor or other health professional that you had hypertension, also called high blood pressure.” Hypertension, measured by the NHANES, was defined as a mean systolic blood pressure ≥ 130 mmHg or diastolic blood pressure or ≥ 80 mmHg, based on four measurements (22). Participants who were diagnosed with diabetes were defined as follows: (1) fasting blood glucose ≥7 mmol/L, (2) Hemoglobin A1c ≥6.5%, (3) self-reported diabetes which was diagnosed by professional physicians before, (4) taking insulin or other hypoglycemic drugs (23, 24). The medical conditions section, identified by the variable name prefix MCQ, includes self-and proxy-reported interview data on a wide range of health conditions and medical history for children and adults. Questions “Has a doctor or other health professional ever informed you that you had congestive heart failure, coronary heart disease, angina, heart attack, stroke, etc.?” (labeled MCQ 160 B-F in household questionnaires administered during home interviews) were utilized to identify participants with a history of CVD if they answered “yes” to any of these questions.

### 2.6 Statistical analysis

Considering the recommendations of NHNAES, appropriate sample weights (SDMVPSU, SDMVSTRA, WTMEC2YR) were applied for all analyses. Continuous variables were expressed as means and standard error (S.E), and weight t-tests were utilized for comparisons between the depression and non-depression groups. The constitutional ratio was provided for categorical variables, and Chi-square tests were employed for comparisons between the two groups. Potential covariates were selected according to the weighted logistic regression models, with detailed results of covariates selection presented in Supplementary Table S1. Weighted multivariable logistic regression models were employed to investigate the associations of eczema, CDAI, with depression symptoms, with results reported as 95% confidence intervals (CIs) and odds ratios (ORs). In addition, the additive interaction between eczema, CDAI, and depression symptoms was measured by relative excess risk due to interaction (RERI) and the attributable proportion of interaction (AP). When the confidence interval of RERI and AP contained 0, there was no additive interaction. Subsequently, the associations of eczema, CDAI, with depression were also explored in different gender, body mass index (BMI), and smoking subgroups. All analyses were conducted using R version 4.2.3 (2023-03-15 ucrt), with a significance threshold of *p* < 0.05.

## 3 Results

### 3.1 Characteristics of participants

**Figure 1:**
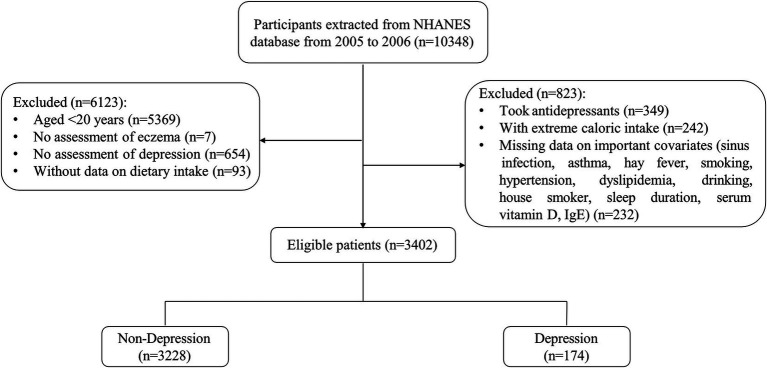
Flowing chart of the selection process.

In total, 3,402 participants were included in the current study (Figure 1). The basic characteristics based on depression were presented in Table 1. Of those participants, the mean age was 46.76 (0.83) years old, and 50.92% were female. And 174 (5.11%) individuals have depression symptoms Statistical differences were found between depression and non-depression groups in PIR, marriage, education level, physical activity, sleep duration, smoking, house smoker, hypertension, diabetes, CVD, serum vitamin D, eczema, CDAI, and six components of CDAI (all *p* < 0.05).

**Table 1:** Characteristics of the participants.

Variables	Total (*n* = 3,402)	Depression		*P*
		No (*n* = 3,228)	Yes (*n* = 174)	
Age, years, mean (±S.E)	46.76 (±0.83)	46.81 (±0.82)	45.70 (±2.10)	0.565 ^a^
Gender, n (%)				0.574 ^b^
Female	1752 (50.92)	1,654 (50.82)	98 (52.97)	
Male	1,650 (49.08)	1,574 (49.18)	76 (47.03)	
Race, n (%)				0.126 ^b^
Non-Hispanic White	1710 (72.45)	1,638 (72.79)	72 (65.00)	
Non-Hispanic Black	762 (11.06)	707 (10.73)	55 (18.32)	
Mexican American	697 (8.03)	659 (7.97)	38 (9.37)	
Other Race	233 (8.46)	224 (8.51)	9 (7.32)	
PIR, n (%)				<0.001 ^b^
<1.85	1,219 (24.57)	1,119 (23.49)	100 (48.29)	
≥1.85	2076 (72.90)	2004 (73.89)	72 (51.30)	
Unknown	107 (2.53)	105 (2.62)	2 (0.42)	
Marriage, n (%)				<0.001 ^b^
Married/Living with partner	2,211 (67.63)	2,118 (68.28)	93 (53.24)	
Single/Separated/Divorced/Widowed	1,191 (32.37)	1,110 (31.72)	81 (46.76)	
Education, n (%)				<0.001 ^b^
Below high school	904 (16.60)	834 (16.12)	70 (27.10)	
High school/GED or Equivale	802 (24.43)	749 (23.88)	53 (36.52)	
Above high school	1,696 (58.97)	1,645 (60.00)	51 (36.39)	
Height, cm, mean (±S.E)	169.03 (±0.24)	169.07 (±0.24)	168.30 (±0.85)	0.374 ^a^
Weight, kg, mean (±S.E)	81.70 (±0.84)	81.60 (±0.83)	84.04 (±2.65)	0.337 ^a^
BMI, kg/m^2^, n (%)				0.919 ^b^
<25	1,024 (33.15)	973 (33.13)	51 (33.66)	
≥25	2,378 (66.85)	2,255 (66.87)	123 (66.34)	
PA, MET·min/week, n (%)				<0.001 ^b^
<450	670 (20.49)	645 (20.69)	25 (16.07)	
≥450	1,433 (47.34)	1,396 (48.36)	37 (24.92)	
Unknown	1,299 (32.17)	1,187 (30.95)	112 (59.01)	
Sleep duration, hours, n (%)				<0.001 ^b^
7–9	2054 (62.80)	1990 (63.85)	64 (39.74)	
<7	1,264 (35.24)	1,159 (34.15)	105 (59.25)	
>9	84 (1.96)	79 (2.00)	5 (1.00)	
Smoking, n (%)				0.001 ^b^
No	1808 (51.88)	1744 (52.92)	64 (29.21)	
Yes	1,594 (48.12)	1,484 (47.08)	110 (70.79)	
Drinking, n (%)				0.400 ^b^
No	476 (11.33)	451 (11.45)	25 (8.61)	
Yes	2,926 (88.67)	2,777 (88.55)	149 (91.39)	
House smoker, n (%)				0.001 ^b^
0	2,801 (82.07)	2,680 (82.73)	121 (67.59)	
1–2	548 (16.18)	501 (15.68)	47 (27.22)	
≥3	53 (1.75)	47 (1.59)	6 (5.19)	
Hypertension, n (%)				0.010 ^b^
No	1,662 (49.93)	1,595 (50.70)	67 (33.06)	
Yes	1740 (50.07)	1,633 (49.30)	107 (66.94)	
Diabetes, n (%)				0.011 ^b^
No	2,971 (90.43)	2,833 (90.75)	138 (83.41)	
Yes	431 (9.57)	395 (9.25)	36 (16.59)	
Dyslipidemia, n (%)				0.504 ^b^
No	968 (29.46)	927 (29.62)	41 (25.93)	
Yes	2,434 (70.54)	2,301 (70.38)	133 (74.07)	
CVD, n (%)				0.002 ^b^
No	3,070 (92.28)	2,928 (92.58)	142 (85.65)	
Yes	332 (7.72)	300 (7.42)	32 (14.35)	
Asthma, n (%)				0.304 ^b^
No	3,003 (87.39)	2,854 (87.54)	149 (83.95)	
Yes	399 (12.61)	374 (12.46)	25 (16.05)	
Hay fever, n (%)				0.334 ^b^
No	3,057 (87.15)	2,899 (86.99)	158 (90.75)	
Yes	345 (12.85)	329 (13.01)	16 (9.25)	
Sinus infection, n (%)				0.112 ^b^
No	2,943 (84.66)	2,803 (85.03)	140 (76.45)	
Yes	459 (15.34)	425 (14.97)	34 (23.55)	
Topical steroids, n (%)				0.461 ^b^
No	3,389 (99.52)	3,215 (99.50)	174 (100.00)	
Yes	13 (0.48)	13 (0.50)	0 (0.00)	
Topical emollients, n (%)				0.829 ^b^
No	3,401 (99.99)	3,227 (99.99)	174 (100.00)	
Yes	1 (0.01)	1 (0.01)	0 (0.00)	
Antihistamines, n (%)				0.805 ^b^
No	3,310 (95.96)	3,142 (95.98)	168 (95.50)	
Yes	92 (4.04)	86 (4.02)	6 (4.50)	
Antipsychotics, n (%)				0.126 ^b^
No	3,386 (99.38)	3,214 (99.46)	172 (97.59)	
Yes	16 (0.62)	14 (0.54)	2 (2.41)	
Energy, kcal, mean (±S.E)	2098.19 (±25.09)	2102.43 (±24.67)	2005.02 (±56.58)	0.066 ^a^
Serum vitamin D, nmol/L, mean (±S.E)	61.11 (±1.22)	61.36 (±1.22)	55.57 (±2.45)	0.020 ^a^
IgE, kU/L, mean (±S.E)	133.51 (±7.75)	133.86 (±8.23)	125.79 (±21.79)	0.750 ^a^
Eczema, n (%)				<0.001 ^b^
No	3,190 (92.67)	3,039 (93.06)	151 (84.06)	
Yes	212 (7.33)	189 (6.94)	23 (15.94)	
CDAI, Mean (±S.E)	0.23 (±0.09)	0.29 (±0.09)	−1.23 (±0.33)	<0.001 ^a^
CDAI, n (%)				0.002 ^b^
High	851 (26.88)	820 (27.48)	31 (13.71)	
Low	2,551 (73.12)	2,408 (72.52)	143 (86.29)	
Vitamin A, mcg, mean (±S.E)	606.11 (±15.10)	610.96 (±14.76)	499.73 (±44.14)	0.016 ^a^
Vitamin C, mg, mean (±S.E)	82.85 (±2.32)	83.90 (±2.28)	59.72 (±7.41)	0.005 ^a^
Vitamin E, mg, mean (±S.E)	7.14 (±0.15)	7.20 (±0.15)	5.87 (±0.42)	0.007 ^a^
Magnesium, mg, mean (±S.E)	290.35 (±2.79)	292.34 (±2.70)	246.65 (±9.46)	<0.001 ^a^
Selenium, mcg, mean (±S.E)	107.83 (±1.57)	108.47 (±1.56)	93.87 (±4.18)	0.002 ^a^
Zinc, mg, Mean (±S.E)	12.41 (±0.31)	12.49 (±0.33)	10.61 (±0.64)	0.019 ^a^

S.E, standard error; ^a^Weighted *t*-test; ^b^Rao-Scott Chi-square test; CDAI, composite dietary antioxidant index; PIR, income-to-poverty ratio; BMI, body mass index; PA, physical activity; CVD, cardiovascular disease; IgE, immunoglobulin E.

### 3.2 Associations of eczema, CDAI, with depression symptoms

The associations of eczema, CDAI, with depression were illustrated in Table 2. After adjusting race, PIR, marriage, education level, sleep duration, smoking, house smoker, hypertension, diabetes, CVD, and serum vitamin D, both eczema (OR = 3.60, 95%CI: 2.39–5.40) and CDAI (OR = 1.97, 95%CI: 1.19–3.27) were associated with higher prevalence of depression symptoms. Compared to the participants with high CDAI and no eczema, those participants with low CDAI (eczema: OR = 7.30, 95%CI: 4.73–11.26; non-eczema: OR = 1.84, 95%CI: 1.06–3.19) have higher odds of depression symptoms, no matter have eczema or not. There was a significant synergistic effect of eczema and low CDAI level on depression symptoms (RERI = 4.22, 95%CI: 0.54–7.90; AP = 0.58, 95%CI: 0.20–0.95). We further explored the association of eczema with depression symptoms under different CDAI levels (Table 3). In individuals with high CDAI levels, no statistical significance relationship was found between eczema and depression symptoms (OR = 2.56, 95%CI: 0.61–10.83). When under low CDAI level, eczema was associated with increased odds of depression symptoms (OR = 3.76, 95%CI: 2.34–6.03).

**Table 2:** The associations of eczema, CDAI with depression symptoms.

Variables	Unadjusted		Model 1	
	OR (95% CI)	*P*	OR (95% CI)	*P*
Eczema				
No	Ref		Ref	
Yes	2.54 (1.82–3.55)	<0.001	3.60 (2.39–5.40)	<0.001
CDAI				
High	Ref		Ref	
Low	2.38 (1.48–3.85)	<0.001	1.97 (1.19–3.27)	0.009
CDAI and Eczema				
Eczema (no) and CDAI (high)	Ref		Ref	
Eczema (no) and CDAI (low)	2.22 (1.31–3.75)	0.003	1.84 (1.06–3.19)	0.030
Eczema (yes) and CDAI (high)	1.51 (0.53–4.29)	0.436	2.24 (0.78–6.41)	0.134
Eczema (yes) and CDAI (low)	6.34 (3.96–10.17)	<0.001	7.30 (4.73–11.26)	<0.001
Measures	Measure (95% CI)		Measure (95% CI)	
RERI	3.62 (0.66–6.57)		4.22 (0.54–7.90)	
AP	0.57 (0.24–0.90)		0.58 (0.20–0.95)	

OR, hazard ratio; CI, confidence intervals; Ref, reference; CDAI, composite dietary antioxidant index; RERI, relative excess risk of interaction; AP, attributable proportion; Unadjusted was crude model; Model 1 adjusting race, PIR, marriage, education, sleep duration, smoking, house smoker, hypertension, diabetes, CVD, and serum Vitamin D.

**Table 3:** The association of eczema with depression symptoms under different CDAI level.

Variables	Unadjusted		Model 1	
	OR (95% CI)	*P*	OR (95% CI)	*P*
CDAI (high)				
No	Ref		Ref	
Yes	1.51 (0.53–4.29)	0.436	2.56 (0.61–10.83)	0.200
CDAI (low)				
No	Ref		Ref	
Yes	2.86 (1.84–4.45)	<0.001	3.76 (2.34–6.03)	<0.001

OR, hazard ratio; CI, confidence intervals; Ref, reference; CDAI, composite dietary antioxidant index; Unadjusted was crude model; Model 1 adjusting race, PIR, marriage, education, sleep duration, smoking, house smoker, hypertension, diabetes, CVD, and serum Vitamin D.

### 3.3 Subgroup analysis

**Figure 2:**
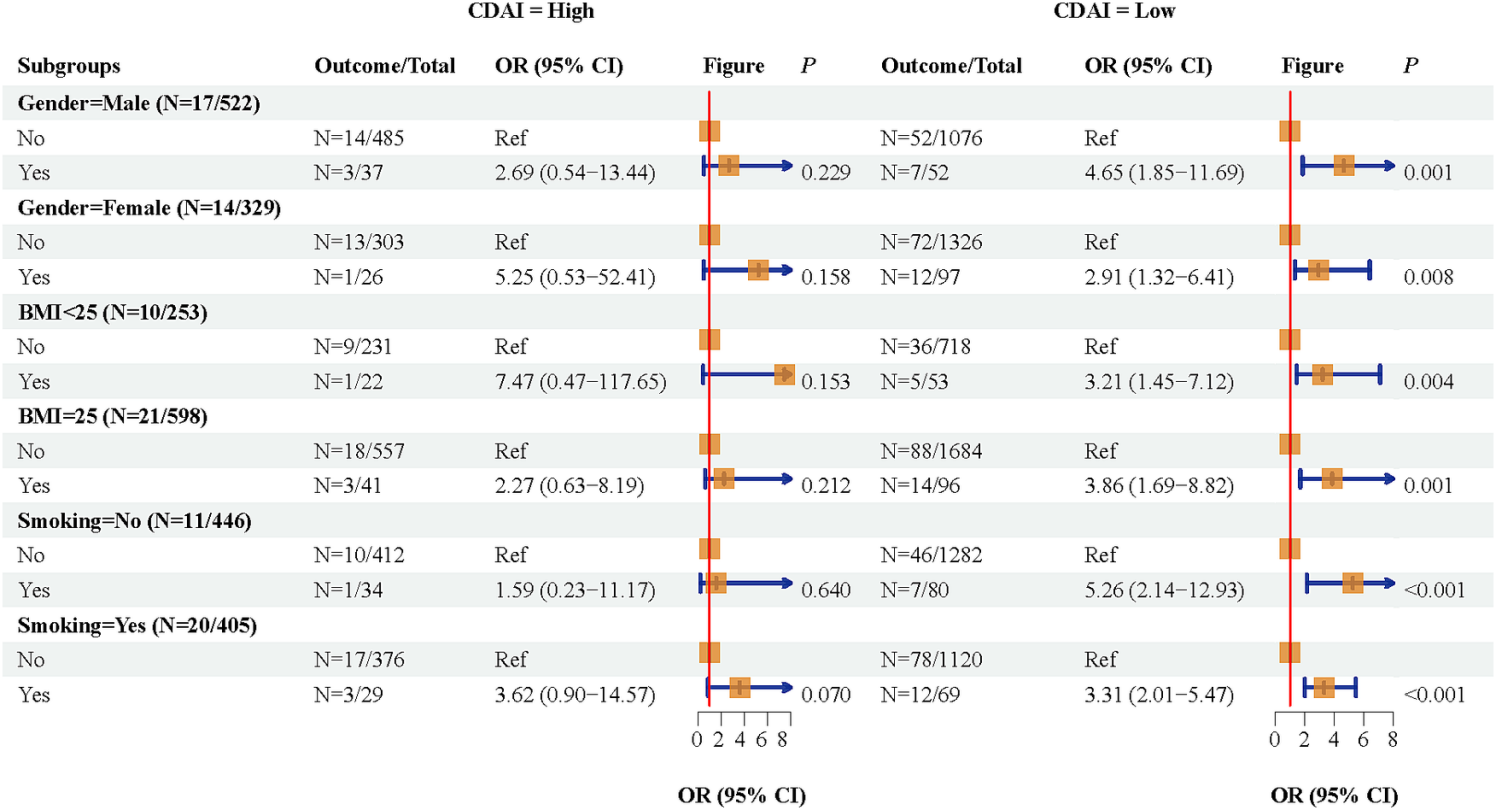
Associations of eczema, CDAI, with depression symptoms in different gender, BMI, smoking subgroups.

The associations of eczema, CDAI, with depression were further explored in various gender, BMI, and smoking subgroups (Table 4) (Figure 2). When under low CDAI level, eczema was related to elevated odds of depression symptoms in those males (OR = 4.65, 95%CI: 1.85–11.69), females (OR = 2.91, 95%CI: 1.32–6.41), BMI <25 (OR = 3.21, 95%CI: 1.45–7.12), BMI ≥25 (OR = 3.86, 95%CI: 1.69–8.82), non-smoking (OR = 5.26, 95%CI: 2.14–12.93), and smoking (OR = 3.31, 95%CI: 2.015.47). When individuals experienced high CDAI levels, no associations were found between eczema and depression symptoms of diverse subgroups. The interaction effect of CDAI and eczema on depression was shown in Table 5.

**Table 4:** The association of eczema with depression symptoms under different CDAI level in subgroups.

Variables	CDAI (high)			CDAI (low)		
	Outcome/Total	OR (95% CI)	*P*	Outcome/Total	OR (95% CI)	*P*
Gender-Male (*n* = 17/522)						
No	*n* = 14/485	Ref		*n* = 52/1076	Ref	
Yes	*n* = 3/37	2.69 (0.54–13.44)	0.229	*n* = 7/52	4.65 (1.85–11.69)	0.001
Gender-Female (*n* = 14/329)						
No	*n* = 13/303	Ref		*n* = 72/1326	Ref	
Yes	*n* = 1/26	5.25 (0.53–52.41)	0.158	*n* = 12/97	2.91 (1.32–6.41)	0.008
BMI <25 (*n* = 10/253)						
No	*n* = 9/231	Ref		*n* = 36/718	Ref	
Yes	*n* = 1/22	7.47 (0.47–117.65)	0.153	*n* = 5/53	3.21 (1.45–7.12)	0.004
BMI ≥25 (*n* = 21/598)						
No	*n* = 18/557	Ref		*n* = 88/1684	Ref	
Yes	*n* = 3/41	2.27 (0.63–8.19)	0.212	*n* = 14/96	3.86 (1.69–8.82)	0.001
Smoking-No (*n* = 11/446)						
No	*n* = 10/412	Ref		*n* = 46/1282	Ref	
Yes	*n* = 1/34	1.59 (0.23–11.17)	0.640	*n* = 7/80	5.26 (2.14–12.93)	<0.001
Smoking-Yes (*n* = 20/405)						
No	*n* = 17/376	Ref		*n* = 78/1120	Ref	
Yes	*n* = 3/29	3.62 (0.90–14.57)	0.070	*n* = 12/69	3.31 (2.01–5.47)	<0.001

**Table 5:** The combined effect of CDAI and eczema on the odds of depression in subgroups.

Subgroups	Outcome/Total	Unadjusted		Model 1	
		OR (95% CI)	*P*	OR (95% CI)	*P*
Gender = Female	*n* = 98/1752				
CDAI and Eczema					
Eczema = No and CDAI=High	*n* = 13/303	Ref		Ref	
Eczema = No and CDAI = Low	*n* = 72/1326	2.12 (0.89–5.07)	0.117	1.94 (0.74–5.05)	0.248
Eczema = Yes and CDAI=High	*n* = 1/26	0.94 (0.12–7.57)	0.954	1.80 (0.25–12.70)	0.588
Eczema = Yes and CDAI = Low	*n* = 12/97	5.37 (2.14–13.46)	0.004	6.06 (2.28–16.13)	0.023
		*P* for trend	0.003	*P* for trend	0.022
RERI		3.31 (−1.42–8.05)		3.33 (−3.09–9.74)	
AP		0.62 (0.09–1.15)		0.55 (−0.17–1.27)	
SI		4.13 (0.31–54.29)		2.92 (0.27–32.13)	
Gender = Male	*n* = 76/1650				
CDAI and Eczema					
Eczema = No and CDAI=High	*n* = 14/485	Ref		Ref	
Eczema = No and CDAI = Low	*n* = 52/1076	2.30 (1.24–4.29)	0.022	1.85 (0.93–3.72)	0.157
Eczema = Yes and CDAI=High	*n* = 3/37	1.95 (0.72–5.24)	0.213	2.42 (0.91–6.41)	0.151
Eczema = Yes and CDAI = Low	*n* = 7/52	8.35 (3.48–20.02)	<0.001	9.61 (3.36–27.52)	0.013
		*P* for trend	<0.001	*P* for trend	0.014
RERI		5.10 (−1.24–11.45)		6.34 (−3.11–15.79)	
AP		0.61 (0.27–0.95)		0.66 (0.27–1.05)	
SI		3.27 (1.03–10.34)		3.79 (0.89–16.09)	
BMI < 25	*n* = 51/1024				
CDAI and Eczema					
Eczema = No and CDAI=High	*n* = 9/231	Ref		Ref	
Eczema = No and CDAI = Low	*n* = 36/718	1.95 (0.80–4.76)	0.167	1.75 (0.73–4.21)	0.276
Eczema = Yes and CDAI=High	*n* = 1/22	0.92 (0.13–6.64)	0.934	1.36 (0.18–10.43)	0.784
Eczema = Yes and CDAI = Low	*n* = 5/53	5.77 (2.20–15.14)	0.004	6.41 (2.34–17.55)	0.022
		*P* for trend	0.005	*P* for trend	0.023
RERI		3.90 (−1.37–9.17)		4.30 (−1.72–10.32)	
AP		0.68 (0.21–1.14)		0.67 (0.18–1.16)	
SI		5.48 (0.29–102.92)		4.87 (0.32–74.43)	
BMI ≥ 25	*n* = 123/2378				
CDAI and Eczema					
Eczema = No and CDAI=High	*n* = 18/557	Ref		Ref	
Eczema = No and CDAI = Low	*n* = 88/1684	2.36 (1.32–4.20)	0.013	1.85 (0.97–3.53)	0.135
Eczema = Yes and CDAI=High	*n* = 3/41	1.95 (0.76–5.01)	0.192	2.45 (0.94–6.36)	0.140
Eczema = Yes and CDAI = Low	*n* = 14/96	6.65 (3.21–13.75)	<0.001	7.54 (3.58–15.88)	0.006
		*P* for trend	<0.001	*P* for trend	0.007
RERI		3.34 (−1.76–8.45)		4.24 (−1.94–10.42)	
AP		0.50 (0.00–1.00)		0.56 (0.06–1.07)	
SI		2.45 (0.64–9.45)		2.84 (0.61–13.24)	
Smoking = No	*n* = 64/1808				
CDAI and Eczema					
Eczema = No and CDAI=High	*n* = 10/412	Ref		Ref	
Eczema = No and CDAI = Low	*n* = 46/1282	2.10 (0.91–4.83)	0.107	1.77 (0.72–4.35)	0.268
Eczema = Yes and CDAI=High	*n* = 1/34	1.29 (0.16–10.34)	0.816	1.53 (0.18–12.78)	0.709
Eczema = Yes and CDAI = Low	*n* = 7/80	7.46 (2.40–23.17)	0.005	9.93 (2.88–34.25)	0.015
		*P* for trend	0.003	*P* for trend	0.014
RERI		5.07 (−2.81–12.95)		7.63 (−3.71–18.96)	
AP		0.68 (0.18–1.18)		0.77 (0.36–1.17)	
SI		4.66 (0.37–58.12)		6.85 (0.36–131.66)	
Smoking = Yes	*n* = 110/1594				
CDAI and Eczema					
Eczema = No and CDAI=High	*n* = 17/376	Ref		Ref	
Eczema = No and CDAI = Low	*n* = 78/1120	2.36 (1.27–4.36)	0.018	1.87 (1.01–3.44)	0.103
Eczema = Yes and CDAI=High	*n* = 3/29	1.64 (0.66–4.03)	0.305	2.51 (1.13–5.57)	0.073
Eczema = Yes and CDAI = Low	*n* = 12/69	6.33 (3.31–12.10)	<0.001	6.62 (3.82–11.46)	0.001
		*P* for trend	<0.001	*P* for trend	0.001
RERI		3.33 (0.17–6.49)		3.24 (−0.31–6.79)	
AP		0.53 (0.22–0.84)		0.49 (0.12–0.86)	
SI		2.67 (1.02–7.00)		2.37 (0.88–6.38)	

OR, hazard ratio; CI, confidence intervals; Ref, reference; CDAI, composite dietary antioxidant index; Model 1 adjusting race, PIR, marriage, education, sleep duration, smoking, house smoker, hypertension, diabetes, CVD, and serum Vitamin D.

## 4 Discussion

Eczema was significantly associated with a higher prevalence of depression symptoms. Similarly, a lower level of CDAI was also associated with a higher prevalence of depression symptoms. Importantly, our study found that low CDAI levels could modulate the relationship between eczema and depression symptoms. Specifically, individuals with both eczema and low CDAI levels exhibited heightened odds of depression compared to those with higher CDAI levels. This indicated the potential protective role of dietary antioxidants in mitigating depressive symptoms associated with chronic inflammatory conditions like eczema.

Our finding was consistent with existing literature reporting the relationship between eczema and depression (7, 25, 26). Chiesa Fuxench et al. (7) reported an increased likelihood of anxiety or depression in patients with eczema, with similar results in Cheng’s research (8). There is a greater prevalence of depression, anxiety, sleep disorders, and suicidal ideation among individuals with eczema (25). Patients with eczema should be vigilant about their mental health. Anyway, our study uniquely contributes by highlighting the role of dietary antioxidants in modifying the association of CDAI with depression symptoms. Previous studies have predominantly focused on the direct impact of inflammatory processes on mental health outcomes, whereas our research underscores the importance of dietary factors in modulating this association (27). Vitamin D and E supplementations demonstrate effectiveness to some extent in improving eczema severity (28). An antioxidant diet is a protective factor against depression, and a negative relationship exists between CDAI and depression (14). Our findings added the reference to the beneficial effect of antioxidants on mental health among patients with eczema.

The mechanisms underlying the moderating of low CDAI levels on the association between eczema and depression symptoms require comprehensive investigation. Dietary antioxidants, such as vitamins C and E, carotenoids, and flavonoids, are essential in neutralizing reactive oxygen species and mitigating inflammation (13). Individuals with eczema experience heightened oxidative stress due to increased inflammation and reactive oxygen species production (1). Oxidative stress was also linked to neuronal damage and altered neurotransmitter function implicated in depression (12). Thus, higher antioxidant intake, as indicated by CDAI, may alleviate oxidative stress in eczema patients, thus reducing depressive symptoms. Secondly, dietary antioxidants possess anti-inflammatory properties that may directly benefit both eczema and depression. Eczema, characterized by skin inflammation, correlates with elevated cutaneous and serum levels of pro-inflammatory cytokines such as interleukin (IL)-4, IL-13, and IL-22 (29). It has been suggested that IL-13, by binding to dopaminergic neurons and stimulating astrocyte production of brain-derived neurotrophic factor, along with oxidative stress, could contribute to neuronal damage in the ventral tegmental and substantia nigra, potentially predisposing individuals to depression and suicidality (30, 31). By mitigating inflammation, antioxidants could potentially mitigate the systemic inflammatory response associated with eczema, potentially easing depressive symptoms. Thirdly, the gut-skin and the gut-brain axes provide additional mechanistic insights. Dietary antioxidants can influence gut microbiota composition and function, thereby modulating immune responses and the production of neuroactive compounds (32). Dysbiosis and impaired gut barrier function observed in eczema patients have been linked to both skin inflammation and depressive symptoms (33). Increased antioxidants intake may restore gut microbial balance, yielding beneficial effects on both eczema severity and mood disorders.

We found that subgroups stratified by gender, BMI, and smoking status consistently demonstrated that low levels of the CDAI intensified the association between eczema and depression symptoms. This suggests that the beneficial effects of dietary antioxidants might be widespread, impacting various demographic groups similarly. Women may experience different hormonal fluctuations that interact with antioxidant levels and their mental health outcomes, while individuals with higher BMI might have altered metabolism affecting the efficacy of antioxidants. Additionally, smoking status could play a crucial role, as smokers often have lower antioxidant levels due to lifestyle factors, potentially exacerbating both eczema and depressive symptoms.

Our findings underscore the significant role that dietary interventions can play in managing mental health outcomes for individuals with eczema. Specifically, healthcare providers should evaluate and optimize antioxidant intake in patients with eczema, as this may help mitigate the risk of depression. We recommend implementing dietary assessments that focus on patients’ current antioxidant consumption. Providers can guide patients toward a diet rich in fruits, vegetables, nuts, and whole grains-key sources of dietary antioxidants-potentially alleviating both eczema symptoms and depressive symptoms. In practical terms, healthcare providers can create structured dietary plans that incorporate these foods, alongside educational resources that highlight the benefits of antioxidant-rich diets. Collaborating with nutritionists or dietitians can further enhance these efforts, providing patients with tailored dietary strategies that align with their preferences and lifestyle constraints. Additionally, addressing the feasibility of such dietary changes is crucial; for instance, practitioners could explore local food resources, budget-friendly options, and simple recipes to make healthier eating more accessible.

The strengths of our study lie in its utilization of a large, nationally representative sample from the NHANES database, facilitating robust statistical analyses. However, several limitations should be acknowledged. Firstly, the cross-sectional design limits our ability to establish causality between CDAI, eczema, and depression. Future longitudinal studies are necessary to clarify the temporal relationships and long-term effects of dietary antioxidants on mental health outcomes. Secondly, dietary intake data were obtained according to the 24-h dietary recall interview, which may introduce potential recall bias. Further research incorporating objective measures of antioxidant status, such as biomarkers, would provide more accurate estimates of dietary antioxidant effects on eczema and depression. Lastly, residual confounding from unmeasured variables in the database cannot be discounted.

## 5 Conclusion

Our study shows that low levels of the CDAI moderate the relationship between eczema and depressive symptoms in U.S. adults. These results suggest that individuals with eczema may experience heightened depressive symptoms when CDAI levels are low. Dietary interventions aimed at increasing antioxidant intake may play a crucial role in the mental health management of individuals with eczema.

## Data Availability

Publicly available datasets were analyzed in this study. This data can be found at: NHANES (https://www.cdc.gov/nchs/nhanes/).
