# Supplementary material for: The impact of Composite Dietary Antioxidant Index on the relationship between eczema and depression symptoms in US adults
Source: Front Nutr. 2024 Nov 22;11:1470833. doi: 10.3389/fnut.2024.1470833 (PMC11620884; doi:10.3389/fnut.2024.1470833)
Supplement: Supplementary file 1 [file Table_1.DOCX]

Table S1 The results of potential covariates selection

| Variables | OR (95% CI) | *P* |
| --- | --- | --- |
| Age | 1.00 (0.98-1.01) | 0.562 |
| Gender |  |  |
| Female | Ref |  |
| Male | 0.92 (0.68-1.23) | 0.565 |
| Race |  |  |
| Non-Hispanic White | Ref |  |
| Non-Hispanic Black | 1.91 (1.20-3.05) | 0.006 |
| Mexican American | 1.32 (0.78-2.21) | 0.297 |
| Other Race | 0.96 (0.35-2.67) | 0.942 |
| PIR |  |  |
| <1.85 | Ref |  |
| ≥1.85 | 0.34 (0.24-0.48) | <0.001 |
| Unknown | 0.08 (0.02-0.35) | 0.001 |
| Marriage |  |  |
| Married/Living with partner | Ref |  |
| Single/Separated/Divorced/Widowed | 1.89 (1.44-2.48) | <0.001 |
| Education |  |  |
| Below High School | Ref |  |
| High School/GED or Equivale | 0.91 (0.57-1.45) | 0.690 |
| Above High School | 0.36 (0.25-0.53) | <0.001 |
| BMI |  |  |
| <25 | Ref |  |
| ≥25 | 0.98 (0.62-1.54) | 0.918 |
| PA |  |  |
| <450 | Ref |  |
| ≥450 | 0.66 (0.40-1.11) | 0.115 |
| Unknown | 2.45 (1.40-4.30) | 0.002 |
| Sleep Duration |  |  |
| 7-9 | Ref |  |
| <7 | 2.79 (1.82-4.26) | <0.001 |
| >9 | 0.81 (0.26-2.49) | 0.709 |
| Smoking |  |  |
| No | Ref |  |
| Yes | 2.72 (1.66-4.48) | <0.001 |
| Drinking |  |  |
| No | Ref |  |
| Yes | 1.37 (0.67-2.82) | 0.388 |
| House Smoker |  |  |
| 0 | Ref |  |
| 1-2 | 2.12 (1.36-3.32) | 0.001 |
| ≥3 | 3.99 (1.55-10.30) | 0.004 |
| Hypertension |  |  |
| No | Ref |  |
| Yes | 2.08 (1.26-3.43) | 0.004 |
| Diabetes |  |  |
| No | Ref |  |
| Yes | 1.95 (1.23-3.10) | 0.005 |
| Dyslipidemia |  |  |
| No | Ref |  |
| Yes | 1.20 (0.71-2.04) | 0.494 |
| CVD |  |  |
| No | Ref |  |
| Yes | 2.09 (1.39-3.13) | <0.001 |
| Asthma |  |  |
| No | Ref |  |
| Yes | 1.34 (0.78-2.32) | 0.289 |
| Hay Fever |  |  |
| No | Ref |  |
| Yes | 0.68 (0.32-1.46) | 0.321 |
| Sinus Infection |  |  |
| No | Ref |  |
| Yes | 1.75 (0.91-3.37) | 0.095 |
| Antihistamines |  |  |
| No | Ref |  |
| Yes | 1.12 (0.45-2.79) | 0.801 |
| Antipsychotics |  |  |
| No | Ref |  |
| Yes | 4.54 (0.61-33.79) | 0.139 |
| Energy | 1.00 (1.00-1.00) | 0.053 |
| Serum Vitamin D | 0.99 (0.97-1.00) | 0.011 |
| IgE | 1.00 (1.00-1.00) | 0.769 |

OR: odds ratio; CI: confidence intervals; Ref: reference;

Age 1.00 (0.98-1.01): 0.996060369247949 (0.982843601546517-1.00945486914217);

Energy 1.00 (1.00-1.00): 0.999838169306117 (0.999674541892893-1.00000182350199);

Serum Vitamin D 0.99 (0.97-1.00): 0.985345861434807 (0.974188985918966-0.996630510794403);

IgE 1.00 (1.00-1.00): 0.999937594945013 (0.999520971867048-1.00035439168096).
